# The impact of tooth loss on cognitive function

**DOI:** 10.1007/s00784-021-04318-4

**Published:** 2021-12-08

**Authors:** Pablo Galindo-Moreno, Lucia Lopez-Chaichio, Miguel Padial-Molina, Gustavo Avila-Ortiz, Francisco O’Valle, Andrea Ravida, Andres Catena

**Affiliations:** 1 Department of Oral Surgery and Implant Dentistry, School of Dentistry, University of Granada, Campus Universitario de Cartuja, s/n, 18071 Granada, Spain; 2 PhD Program in Clinical Medicine and Public Health, University of Granada, Granada, Spain; 3 Department of Periodontics, University of Iowa College of Dentistry, Iowa City, IA USA; 4 Department of Pathology and IBIMER, School of Medicine, University of Granada, Granada, Spain; 5 Instituto Biosanitario de Granada (Ibs.GRANADA), University of Granada, Granada, Spain; 6 Department of Periodontics and Oral Medicine, School of Dentistry, University of Michigan, Ann Arbor, MI USA; 7 Department of Experimental Psychology, School of Psychology, University of Granada, Granada, Spain

**Keywords:** Oral health, Mastication, Cognitive dysfunction, Dementia, Aging, Dental prosthesis

## Abstract

**Objective:**

To investigate if there is epidemiological evidence of an association between edentulism and cognitive decline beside that currently available from limited sample-sized case series and cross-sectional studies considering limited co-variables.

**Materials and methods:**

Data from two USA national health surveys [NHIS 2014–2017 and NHANES 2005–2018] were analyzed using multinomial logistic regression to study the impact of type of edentulism and number of remaining teeth on memory and concentration problems. Age, gender, socioeconomic status, education level, cardiovascular health index, body mass index, exercise, alcohol, smoking habits, and anxiety and depression were used as covariates.

**Results:**

The combined population sample was 102,291 individuals. Age, socioeconomic status, educational level, anxiety and depression levels, and edentulism showed the highest odds ratios for cognitive decline. Number of teeth present in the mouth was found to be a predictor of cognitive status. This association showed a gradient effect, so that the lower the number of teeth, the greater the risk of exhibiting cognitive decline.

**Conclusions:**

Edentulism was found among the higher ORs for cognitive impairment.

**Clinical relevance:**

Maintenance of functional teeth through the promotion of oral health may contribute to the preservation of memory/concentration and other essential cognitive functions. Thus, increasing and efficiently coordinating efforts aimed at preventing of tooth loss in the adult population could substantially contribute to reduce the incidence of cognitive impairment.

**Supplementary Information:**

The online version contains supplementary material available at 10.1007/s00784-021-04318-4.

## Introduction

Oral diseases rank among some of the most prevalent health conditions worldwide [1] affecting approximately 3.6 billion people, with edentulism accounting for 18.45 million global incidences [2]. Edentulism is a major cause of global productivity losses (US$127 billion), followed by periodontitis (US$38 billion) and caries (US$22 billion) [3]. Periodontitis and edentulism have been associated with numerous systemic health disorders [4], including mental health conditions, such as depression, anxiety, or alterations in personality traits (e.g. emotional stability and consciousness) [5].

Neurological disorders are the leading cause of disability-adjusted life-years (DALY) globally [6]. This has in fact increased in most world regions in the last 20 years. Of those disorders, the global impact of neurocognitive disorders (NCDs) is also expected to escalate worldwide in parallel with longevity, from 44 million people in 2013 to 76 million in 2030 and 131 million by 2050 [7]. The socioeconomic impact of this trend is expected to be higher in low- and middle-income countries [8], and will have a total estimated cost of around 1 trillion US dollars by 2018 and 2 trillion by 2030 [9], with a mean cost per person of US $43,680 in G7 countries and US $20,187 in G20 countries [10].One of the most common NCDs is mild cognitive impairment (MCI) [11]. MCI is a syndrome defined as cognitive decline greater than that expected for an individual’s age and education level but that does not interfere notably with activities of daily life, and may be indicative of Alzheimer’s disease or another dementia [12]. Alzheimer’s disease and other types of dementia are the second group of disorders to which DALYs are risk attributable, only after stroke [6]. Among such risks factors, the 2020 report of the Lancet Commission for Dementia proposed three new risk factors, namely, alcohol intake, head injury, and air pollution, in addition to the nine previously proposed in 2017 (i.e., less education, hypertension, hearing impairment, smoking, obesity, depression, physical inactivity, diabetes, and infrequent social contact) [13]. By tackling them, about 40% of dementia cases may be potentially prevented [13, 14]. It is interesting, though, that among such modifiable factors, oral health and edentulism are not included.

The possible etiologic role of edentulism in the development of MCI and dementia has attracted the attention of researchers in recent years [15–17]. Possible mechanisms supporting this relationship, as reviewed elsewhere [18], include the following: (1) the inflammation/infection mechanism: *Porphyromonas gingivalis*, a well-known periodontopathogen, may induce the local release of pro-inflammatory cytokines [19] and subsequently increase both the peripheral circulation [20] and brain accumulation of amyloid-β [21]; (2) the masticatory mechanism: preclinical and clinical studies have demonstrated the negative effect of impaired masticatory function on the incidence of cognitive performance [22]; (3) the diet and nutrition mechanism: this is strongly related to the masticatory one, since tooth loss may influence dietary patterns, which can have a deleterious effect on intraoral food preprocessing before deglutition, leading to a reduction in the intake of nutritional components that have a neuroprotective effect and also promote obesity [23].

However, the heterogeneity of studies, the different outcome measures for cognitive decline, and the variable inclusion of important confounders across the studies make it difficult to clearly estimate an effect size in this association. Furthermore, most studies only evaluate the number of missing teeth, whereas mastication is a multifaceted functional activity that cannot be measured on the basis of a mere numeric value. Little is known about the “larger picture;” particularly, the impact that all-cause edentulism may have on cognitive function in diverse populations from different cultural backgrounds. Hence, the aim of this study was to investigate the association between edentulism and cognitive function through a combined analysis of large national health surveys. We hypothesized that cognitive function depends, at least in part, on tooth status and masticatory function when other potential important factors, such as age or socioeconomic status, are controlled.

## Material and methods

### Design and sample

This is a retrospective observational study in which data from two national health surveys were analyzed to assess the potential association between oral health and cognitive status. The United States of America’s (USA) National Health Interview Survey (NHIS, 2014–2017) and the National Health and Nutrition Examination Survey (NHANES, 2005–2018) were used. These are public databases and no ethics approval is required to use their data. They both measured the cognitive status (memory/concentration problems) subjectively. The NHIS asked the participants four questions relevant to understand their cognitive function: (1) degree of difficulty remembering or concentrating? (no difficulty, some, a lot, cannot do at all), (2) difficulty remembering, concentrating, or both? (remembering only, concentrating only, both), (3) how often do you have difficulty remembering? (sometimes, often, all the time), and (4) number of things you have difficulty remembering? (a few, a lot, almost everything). This survey also presents some limitations regarding dental status, as only either presence of complete adult dentition or absence of at least one tooth was registered. The NHANES survey asked the participants only a cognitive question (memory/concentration problems), but included an examination of the dentition status with detailed information regarding each individual tooth. Both surveys collected information about age, sex, education level, socioeconomic status (SES), body mass index.

### Outcomes

The primary outcome was cognitive function relative to edentulism in adults 45 years or older. Age, gender, SES, education level, cardiovascular health index, body mass index, exercise, alcohol and smoking habits were used as covariates. In the NHIS analysis, we also included depression and anxiety as confounders. In the NHANES analysis, only depression was included, as the survey did not ask questions about anxiety. Variables and confounders included in this study were coded in an attempt to homogenize the data, as shown in Supplementary Tables 1 and 2. Therefore, the independent variable was edentulism as measured in each survey, and the dependent variables were the cognitive function measures, as defined in each survey as well.

### Statistical analysis

Nominal regression (logistic multinomial) was used to analyze the impact of edentulism and oral hygiene habits on cognitive function. The robustness of the findings respective to the missing values was tested using two regression models, one with covariates (model 1, being the reference category the last one on each variable), and one in which the confounders were excluded (model 2). In all the analyses, odd ratios were calculated using complete natural adult dentition as the reference for edentulism, and “no difficulty at all” for cognitive variables, so that, *OR* > 1 indicated higher probability of having cognitive problems in the edentulous compared to non-edentulous participants. The statistical significance level was set at 0.05. For the NHANES survey, the number of remaining teeth was subjected to an ROC curve analysis, being the state variable “memory/concentration problems.” Using the Unal method [24], the best cut-off point was 20.5 teeth. Additionally, the number of molars, premolars, incisors, and canines was counted in order to determine the relative influence of each tooth type on memory/concentration problems. Again, using the Unal method, the best cut-off points were 5.5, 5.5, 3.5, and 4.5, respectively, for molars, premolars, canines, and incisors. Analyses were conducted using IBM SPSS, version 24 (IBM, Armonk, NY, USA).

## Results

The results of the analysis of USA NHIS data are displayed in Tables 1 and 2. The sample was composed by 17,134 (1169 missing) in 2014, 15,075 (1864 missing) in 2015, 15,350 (1128 missing) in 2016, and 12,122 (1203 missing) in 2017. All cases for which all the variables of interest were measured were included in the model. Therefore, no exclusion criteria were used. Table 1 shows the results of the analysis of 2014 to 2017 data relative to the cognitive difficulties questions related to difficulty with memory or concentration. It was observed that the presence of teeth is beneficial in the maintenance of a good cognitive status. Absence of edentulism is associated with *ORs* < 1 in the five pooled surveys for the categories “some difficulty” and “a lot of difficulty,” respective to the reference category “no difficulty.” These results were similar for model 2, without covariates, although, as expected, were lower than for model 1. Some other variables also appeared to affect cognitive function. As shown in Table 2, and as expected, age, education level, or SES, together with cardiovascular health, exercise, and anxiety and depression, had the most significant OR.

**Table 1:** Multinomial logistic regression effects of edentulism on cognitive function in the NHIS survey. Model 1 is a model with covariates and model 2 is the regression model without covariates

	Edentulism (model 1)		Edentulism (model 2)	
	*OR*	95% CI	*OR*	95% CI
Cognitive status				
Some	1.24	[1.15 1.33]	1.82	[1.72 1.94]
A lot	1.29	[1.10 1.51]	2.67	[2.35 3.02]
Cognitive problem				
Remember	1.20	[1.09 1.33]	1.81	[1.66 1.96]
Concentrating	1.22	[1.00 1.50]	1.66	[1.40 1.97]
Both	1.30	[1.18 1.42]	2.02	[1.88 2.17]
Remembering frequency				
Sometimes	1.21	[1.12 1.31]	1.76	[1.65 1.88]
Often	1.31	[1.16 1.48]	2.31	[2.10 2.54]
Remembering amount				
A few things	1.22	[1.13 1.32]	1.75	[1.64 1.87]
A lot	1.32	[1.16 1.51]	2.60	[2.33 2.89]

Cognitive status model 1 Nagelkerke *R*^2^ = 0.23; cognitive problem, 0.22; remembering frequency, 0.21; remembering amount, 0.21

**Table 2:** Multinomial logistic regression effects of the control variables on cognitive function in the NHIS surveys

	Sex		Age		BMI		Education		SES		Oral health	
	*OR*	95% CI	*OR*	95% CI	*OR*	95% CI	*OR*	95% CI	*OR*	95% CI	*OR*	95% CI
Cognitive status												
Some	1.09	[1.03 1.14]	2.00	[1.89 2.12]	0.95	[0.93 0.97]	0.86	[0.82 0.91]	1.34	[1.28 1.40]	1.02	[0.99 1.06]
A lot	0.87	[0.77 0.99]	2.20	[1.90 2.53]	0.88	[0.83 0.93]	0.67	[0.60 0.76]	1.40	[1.28 1.53]	1.09	[1.02 1.17]
Cognitive problem												
Remember	1.09	[1.02 1.18]	2.57	[2.38 2.77]	0.94	[0.91 0.97]	0.89	[0.83 0.95]	1.27	[1.20 1.36]	1.03	[0.99 1.07]
Concentrating	0.96	[0.83 1.11]	1.22	[1.03 1.45]	0.96	[0.9 1.03]	0.88	[0.77 1.02]	1.26	[1.12 1.40]	1.04	[0.95 1.13]
Both	1.07	[1.00 1.14]	1.70	[1.58 1.83]	0.94	[0.91 0.97]	0.79	[0.74 0.85]	1.40	[1.33 1.47]	1.03	[0.99 1.07]
Remembering frequency												
Sometimes	1.13	[1.07 1.19]	2.07	[1.95 2.21]	0.95	[0.92 0.97]	0.85	[0.81 0.90]	1.30	[1.23 1.36]	1.03	[0.99 1.07]
Often	0.94	[0.85 1.03]	2.11	[1.90 2.34]	0.92	[0.88 0.95]	0.80	[0.73 0.87]	1.45	[1.36 1.56]	1.02	[0.97 1.08]
Remembering amount												
A few things	1.12	[1.06 1.18]	2.06	[1.94 2.19]	0.95	[0.92 0.97]	0.86	[0.81 0.91]	1.32	[1.26 1.38]	1.01	[0.98 1.05]
A lot	0.88	[0.79 0.98]	2.21	[1.96 2.50]	0.91	[0.86 0.95]	0.75	[0.68 0.83]	1.41	[1.30 1.53]	1.11	[1.05 1.18]
	Alcohol		Smoking		CV health		Exercise		Anxiety		Depression	
	*OR*	95% CI	*OR*	95% CI	*OR*	95% CI	*OR*	95% CI	*OR*	95% CI	*OR*	95% CI
Cognitive status												
Some	0.97	[0.93 1.00]	1.05	[1.01 1.09]	1.24	[1.21 1.27]	0.94	[0.91 0.97]	0.74	[0.72 0.75]	0.71	[0.7 0.73]
A lot	0.82	[0.76 0.89]	1.11	[1.02 1.20]	1.31	[1.25 1.39]	0.71	[0.65 0.77]	0.65	[0.61 0.68]	0.54	[0.51 0.57]
Cognitive problem												
Remember only	1.01	[0.97 1.06]	1.04	[0.99 1.10]	1.23	[1.20 1.27]	0.94	[0.90 0.99]	0.82	[0.79 0.85]	0.79	[0.76 0.82]
Concentrating only	0.82	[0.74 0.90]	1.05	[0.95 1.15]	1.11	[1.04 1.18]	0.97	[0.89 1.06]	0.68	[0.64 0.73]	0.68	[0.63 0.72]
Both	0.93	[0.89 0.97]	1.06	[1.01 1.11]	1.29	[1.25 1.33]	0.89	[0.85 0.93]	0.67	[0.65 0.69]	0.64	[0.62 0.66]
Remembering frequency												
Sometimes	1.00	[0.96 1.03]	1.03	[0.99 1.07]	1.25	[1.22 1.28]	0.96	[0.92 0.99]	0.77	[0.75 0.79]	0.75	[0.75 0.79]
Often	0.90	[0.85 0.96]	1.12	[1.05 1.19]	1.29	[1.24 1.34]	0.78	[0.74 0.83]	0.66	[0.64 0.69]	0.62	[0.59 0.64]
Remembering amount												
A few things	1.01	[0.97 1.05]	1.05	[1.01 1.09]	1.25	[1.22 1.28]	0.95	[0.92 0.99]	0.76	[0.74 0.78]	0.74	[0.72 0.76]
A lot/almost everything	0.81	[0.76 0.87]	1.05	[0.98 1.13]	1.30	[1.24 1.36]	0.73	[0.68 0.79]	0.66	[0.63 0.69]	0.59	[0.56 0.62]

Table 1 shows the results of analyses aimed at discriminating the effect of edentulism on the type of cognitive difficulty (i.e., remembering, concentrating, or both). Relative to the reference category “no problem,” completely edentulous subjects exhibited a larger *OR* for having difficulties in remembering only, concentrating only and both. This was also observed for gender, education, SES, and CV risks variables, among others (Table 2).

Additional data analyses relative to the frequency of memory failures were conducted (Table 1 and Table 2). Relative to the reference category “no problem,” it was found that completely edentulous subjects have an increased probability of memory failures either “sometimes,” “often,” or “all the time.” This was also observed in relation to SES (strongest association), gender, education, smoking, and CV risks.

After quantification of memory failures in function of edentulism, it was found that, relative to the reference category “no problem,” completely edentulous subjects exhibited an increased probability of forgetting “a few things” and “a lot of things” (Table 1 and Table 2). The same trend was observed for age, education, SES, cardiovascular health, exercise, and anxiety and depression.

Data from the NHANES surveys (*N* = 17,189, missing = 5061, overall) were used to precisely analyze the impact of tooth loss on the development of cognitive problems (Table 3, see Supplementary Table 2). The ROC curve analysis indicated that the best cut-off was 20.5 remaining teeth. Results showed that, compared with no edentulism (more than 20.5 teeth), edentulism has a significantly higher probability of being associated with confusion/memory problems (Table 3). When analyzed independently by tooth type, the worst results emerged when molars are missing. Note that absence of molars, but not absence of any other tooth type, is determinant of the effects on memory/confusion problems. All the control variables have also an effect, except for oral health and hypertension. We also tested whether people under age 45 showed an increased probability of having memory/confusion problems. We found an *OR* = 1.877 (95% CI [1.331, 2.646]).

**Table 3:** Multinomial logistic regression effects of edentulism and confounders in the NHANES surveys. The last four rows show the effects of dentition status according to their position in mouth on the cognitive function. Model 1 is the model with the covariates and Model 2 is a model without covariates

	Model 1		Model 2	
Variable	*OR*	95% CI	*OR*	95% CI
Sex	0.76	[0.64 0.90]		
Age	1.43	[1.18 1.72]		
BMI	0.87	[0.79 0.96]		
Education	0.89	[0.83 0.95]		
SES	1.23	[1.16 1.32]		
Oral health	1.01	[0.98 1.03]		
Alcohol	1.17	[1.04 1.30]		
Smoking	1.01	[0.92 1.11]		
CV health	1.24	[1.15 1.35]		
Hypertension	0.97	[0.82 1.15]		
Exercise	0.73	[0.63 0.85]		
Depression	0.87	[0.86 0.88]		
Edentulism	1.22	[1.02 1.45]	2.38	[2.19 2.59]
Molars	1.32	[1.05 1.66]	1.50	[1.33 1.70]
Premolars	1.07	[0.84 1.36]	1.37	[1.20 1.55]
Canines	0.84	[0.65 1.09]	1.12	[0.98 1.28]
Incisors	1.06	[0.81 1.38]	1.34	[1.17 1.55]

Model 1 Nagelkerke *R*^2^ = 0.23

## Discussion

In summary, the results from this survey data analysis study indicate that edentulism is correlated with a decline in cognitive function. Our findings, which are based on a large sample size, support this notion beyond the effect of other epidemiological variables, such as age and current SES. To the best of our knowledge, this is the largest study to date that proves this relationship.

Previous studies have indicated that the number of remaining teeth is associated with cognitive function [25–28]. In our study, the cut-off value was established at 20 teeth. This means that cognitive function is likely to be affected even when only 8 teeth out of a total of 28, not including third molars, are missing. Thus, preservation of all functional teeth is of paramount importance, not only for oral health but also for cognitive function.

Other investigations have concluded that the impact on cognitive function is not influenced by the number of teeth lost per se, but rather results from loss of masticatory function [29]. In this sense, it is known that molars are the teeth that support more masticatory force and mainly determine masticatory efficiency, both in natural and prosthetic occlusion [30]. Thus, masticatory function with either natural teeth or a prosthetic rehabilitation may positively influence cognitive function [31]. In fact, recent data from large-scale European surveys suggests that patients who had prosthetic replacement of missing teeth may exhibit better cognitive performance in comparison to those with no tooth replacement [32].

More interestingly, when analyzed independently by type of tooth missing, the only significant association between cognitive decline and tooth loss was observed when molars are absent. This could be channeled through the Locus Coeruleus that is activated, among others, by the periodontal fibers and proprioceptive jaw muscle spindles [18]. Even more, the activation of the most important and potent masticatory muscle related to the activity of the molar teeth, the masseter, could help release important neurotrophic factors, such as brain-derived neurotrophic factor (BDNF), through the release of cathepsin B and irisin [18]. This effect might be similar as that of exercise [33].

Cognitive problems are generally more frequent and debilitating in the elderly. In our analysis, as expected, older age was strongly associated with the development of cognitive impairment. It is important to remark that the prevalence of periodontitis also increases with age [34]. The analysis of data from the NHIS survey revealed that early tooth loss in people under 45 years of age was associated with impaired cognitive function. Therefore, if we were to hypothesize a time frame for this association, it could be proposed that effective strategies to promote oral health and dental maintenance in all age groups are important to minimize the impact of edentulism on cognitive function [35].

SES also seems to be an important factor in cognitive impairment. The relationship between SES and access to dental healthcare has been widely discussed in the literature [36]. Better and more efficient healthcare is usually available to individuals with higher income, which may partially explain a lower prevalence of cognitive impairment in these groups [37]. Furthermore, it has been shown that a higher SES during childhood favors brain and cognitive development in the long-term [38].

Several mechanisms have been proposed in the literature to explain the association between edentulism and NCDs. Beside non-modifiable risks factors, such as age or sex, social (e.g., network activity) and behavioral habits (e.g., smoking, diet and nutrition, exercise), chronic systemic conditions (e.g., obesity, diabetes, hypertension, hypercholesterolemia), as well as emotional (e.g., depression) and cognitive elements (e.g., level of education, cognitive training), have been linked with NCDs through physiologic alterations that can affect the central nervous system due to an increase in inflammation, vascular damage, oxidative stress, and neurotoxicity [13]. Interestingly, many of these factors are also involved in the etiopathogenesis of periodontitis [39],which may partially explain the link between NCDs and complete or partial edentulism associated with a history of severe periodontitis (stage III or IV).

Substantial efforts have been and are currently being made by governmental agencies, institutions, and researchers to prevent and minimize the deleterious effects of cognitive decline on the global population. Our findings suggest that tooth loss is strongly associated with reduced cognitive function. Hence, it is plausible that by increasing the efforts dedicated to the prevention of tooth loss in the adult population, which would equate to much lower than that dedicated to treat cognitive loss, the overall costs of treating such conditions would be significantly reduced. Delaying the establishment of cognitive impairment by only one year would result in saving approximately €375 billion in Europe alone; fourfold less than the total budget dedicated to dental healthcare, estimated to be 93 thousand million euros in 2020 [40].

Our results also emphasize the importance of oral health and remaining functional teeth in the maintenance of memory. Aside from economic ramifications, the real impact of loss of cognitive function, if we consider this as the loss of memory, as well as personal experiences and interactions, is not truly measurable. Thus, by preventing tooth loss, it may be possible to preserve and protect other aspects of an individual’s well-being that bear no quantitative measure: the ability to live independently, preserve memories, and maintain personal self-identity.

Strengths of this study are the use of large surveys that combined the magnitude of the sample size, which included individuals with diverse background features, supports the generalizability of our findings, and the application of robust statistical methods to assess outcomes, predictors, and confounders. The main limitation was the inability to determine whether a causal relationship between edentulism and cognitive functions exists, due to the cross-sectional nature of the surveys included in the study. Another important limitation of the dataset was that the reason for tooth loss (e.g., periodontitis, caries, trauma) was not reported. It would have been interesting to factor that in the statistical analyses. It is also important to keep in mind the limitation in the evaluation of the actual dental status in the NHIS survey, which registered the “presence of complete adult dentition or absence of at least one tooth.” Meanwhile, the NHANES survey asked for the presence/absence of each tooth; in contrast, cognitive evaluation in the NHANES survey is limited to a single question on memory/confusion. Thus, future prospective observational studies involving large and diverse populations and solving those issues are warranted to investigate a possible etiological connection between edentulism and NCDs, as well as the role that concomitant social and systemic factors may play in this binomial relationship. Furthermore, experimental research is needed to disentangle the crucial causal relationship by addressing the following key question: “Does edentulism lead to poorer cognition or rather poor cognition leads to edentulism?”. Preclinical studies investigating the effect that early tooth loss has on cognitive function may contribute to shed light on this matter. Investigations assessing whether rehabilitation of the masticatory function can enhance and maintain the cognitive status are also warranted. The current lack of knowledge in this cause-effect association likely derives from the fact that there is a paucity of well-conducted prospective clinical studies investigating the association between edentulism and cognition. In our assessment of the literature in this particular topic, we have been able to identify only one pilot study [41], and two protocols for clinical studies, one set in Sweden published in 2021 [42] and the other one, registered in 2016 and still ongoing, by our own group in Spain [43].

## Supplementary Information

Below is the link to the electronic supplementary material.

Supplementary file1 (DOCX 40 KB)
