# Supplementary material for: The impact of tooth loss on cognitive function
Source: Clin Oral Investig. 2021 Dec 8;26(4):3493–500. doi: 10.1007/s00784-021-04318-4 (PMC8979879; doi:10.1007/s00784-021-04318-4)
Supplement: Supplementary file 1 — Supplementary file1 (DOCX 40 KB) [file 784_2021_4318_MOESM1_ESM.docx]

# The Impact of Tooth Loss on Cognitive Function

Pablo Galindo-Moreno, DDS, PhD^1^; Lucia Lopez-Chaichio, DDS^1,2^; Miguel Padial-Molina, DDS, PhD^1^; Gustavo Avila-Ortiz, DDS, MS, PhD^3^; Francisco O´Valle, MD, PhD^4,5^; Andrea Ravida, DDS^6^; Andres Catena, PhD^7^.

^1^ Department of Oral Surgery and Implant Dentistry, School of Dentistry, University of Granada, Granada, Spain.

^2^ PhD Program in Clinical Medicine and Public Health, University of Granada.

^3^ Department of Periodontics, University of Iowa College of Dentistry, Iowa City (IA), USA.

^4^ Department of Pathology and IBIMER, School of Medicine, University of Granada.

^5^ Instituto Biosanitario de Granada (ibs.GRANADA), University of Granada.

^6^ Department of Periodontics and Oral Medicine, School of Dentistry, University of Michigan, Ann Arbor (MI), USA.

^7^ Department of Experimental Psychology, School of Psychology, and Mind, Brain and Behavior Research Center, University of Granada.

**Corresponding author**:

Pablo Galindo-Moreno

School of Dentistry, Campus Universitario de Cartuja, s/n

18071, Granada, Spain

Phone: (+34) 958 249032

Email: pgalindo@ugr.es

# Supplementary Tables

**Supplementary Table 1. Codification of the NHIS surveys.**

| Variable | Values | Labels | N | Source | Questions |
| --- | --- | --- | --- | --- | --- |
| Age | 1 | <45 y | 24668 | AGE_P (SAF) | Age |
|  | 2 | 45 : 70 y | 25734 |  |  |
|  | 3 | >70 y | 9279 |  |  |
| Gender | 1 | Male | 27098 |  | Gender |
|  | 2 | Female | 32583 |  |  |
| Education | 1 | Elementary | 7626 | EDUC1 (SPF) | Highest level of school completed |
|  | 2 | Secondary | 45151 |  |  |
|  | 3 | University | 6904 |  |  |
| Edentulism | 1 | Complete denture | 54520 | LUPPRT (SAF) | Lost all upper & lower natural teeth |
|  | 2 | Edentulism | 5161 |  |  |
| Cognitive Status | 1 | No difficulty | 49795 | COG_SS (FDF) | Degree of difficulty remembering or concentrating |
|  | 2 | Some difficulty | 8572 |  |  |
|  | 3 | A lot of diff/Cannot' do | 1311 |  |  |
| Cognitive Problem | 1 | No problem | 49795 | COG_1 (FDF) | Difficulty remembering, concentrating, or both? |
|  | 2 | Difficulty remembering only | 3285 |  |  |
|  | 3 | Difficulty concentrating only | 1254 |  |  |
|  | 4 | Bot remembering/concentrating | 5333 |  |  |
| Remembering Frequency | 1 | Never | 51049 | COG_2 (FDF) | How often have difficulty remembering? |
|  | 2 | Sometimes | 6204 |  |  |
|  | 3 | Often/All the time | 2417 |  |  |
| Remembering Amount | 1 | Nothing | 51049 | COG_3 (FDF) | Amount of things you have difficulty remembering? |
|  | 2 | A few things | 6927 |  |  |
|  | 3 | A lot of/Almost everything things | 1690 |  |  |
| BMI | 1 | Underweight (<18.5) | 12963 | BMI (SAF) | Body Mass Index (BMI) |
|  | 2 | Normoweight (18.5 : 24.99) | 15541 |  |  |
|  | 3 | Overweight (25 : 29.99) | 15881 |  |  |
|  | 4 | Obesity (>30) | 15296 |  |  |
| Alcohol | 1 | Never drink | 11775 | ALCSTAT (SAF) | Alcohol drinking status: Recode |
|  | 2 | Former drinker | 8939 |  |  |
|  | 3 | Current drinker | 38967 |  |  |
| Smoking | 1 | Never smoke | 35897 | SMKSTAT2 (SAF) | Smoking Status: Recode |
|  | 2 | Former smoker | 14204 |  |  |
|  | 3 | Current smoker | 9580 |  |  |
| CV Risks | 0 | No risks | 30607 | HYPEV (SAF) | Ever been told you have hypertension |
|  | 1 | One risk | 14834 | CHLEV (SAF) | Ever told you had high cholesterol |
|  | 2 | Two risks | 9035 | CHDEV (SAF) | Ever been told you had coronary heart disease |
|  | 3 | Three risks | 4061 | STREV (SAF) | Ever been told you had a stroke |
|  | 4 | Four or more risks | 1144 | DIBEV (SAF) | Ever been told that you have diabetes |
| SES | 0 | Good (0 problems) | 49514 | AHCAFYR1 (SAF) | Couldn't afford prescrip medicine, past 12 m |
|  | 1 | So so (1 to 3 problems) | 7369 | AHCAFYR2 (SAF) | Couldn't afford mental health ..., past 12 m |
|  | 2 | Bad (>3 problems) | 2798 | AHCAFYR3 (SAF) | Couldn't afford dental care, past 12 m |
|  |  |  |  | AHCAFYR4 (SAF) | Couldn't afford eyeglasses, past 12 m |
|  |  |  |  | AHCAFYR5 (SAF) | Couldn't afford to see a specialist, past 12 m |
|  |  |  |  | AHCAFYR6 (SAF) | Couldn't afford follow-up care, past 12 m |
| Exercise | 1 | A lot | 16991 | VIGFREQW (SAF) | Vigorous physical activity |
|  | 2 | Normal | 17364 | MODFREQW (SAF) | Moderate physical activity |
|  | 3 | Few | 25326 | STRFREQW (SAF) | Strengthen physical activity |
| Anxiety | 1 | Very high | 5445 | ANX_1 (FD) | How often feel worried, nervous, or anxious? |
|  | 2 |  | 6300 | ANX_2 (FD) | Take medication for worried, or anxious feeling? |
|  | 3 |  | 5257 | ANX_3R (FD) | Level of feelings when last felt worried, nervous, or anxious? |
|  | 4 |  | 17890 |  |  |
|  | 5 | No | 24789 |  |  |
| Depression | 1 | Very high | 2331 | DEP_1 (FD) | How often do you feel depressed? |
|  | 2 |  | 3008 | DEP_2 (FD) | Take medication for depression? |
|  | 3 |  | 3517 | DEP_3R (FD) | How depressed you felt last time you were depressed? |
|  | 4 |  | 14197 |  |  |
|  | 5 | No | 36628 |  |  |

Note: SAF: Sample Adult File; FD: Functional and Disability File; SPF: Sample Personal File. N: valid sample size for the category.

**Supplementary Table 2. Codes of the NHANES surveys.**

| Variable | Values | Labels | | N | Source | Questions | |  |
| --- | --- | --- | --- | --- | --- | --- | --- | --- |
| Age | 1 | 45 : 70 y | | 15869 | RIDAGEYR | Age in years at screening | |  |
|  | 2 | >70 y | | 6381 |  |  | |  |
| Gender | 1 | Male | | 10948 | RIAGENDR | Gender | |  |
|  | 2 | Female | | 11302 |  |  | |  |
| Education | 1 | Elementary | | 6405 | DMDEDUC2 | Education level - Adults 20+ | |  |
|  | 2 | Secondary | | 11048 |  |  | |  |
|  | 3 | University | | 4751 |  |  | |  |
| Edentulism | 1 | No (>20.5 teeth) | | 11517 | OHX01TC to OHX32TC | | Tooth Counts | |
|  | 2 | Yes (<=20 teeth) | | 9828 |  | |  | |
| Memory | 1 | Yes | | 2755 | PFQ057 | Experience confusion/memory problems? | |  |
|  | 2 | No | | 19481 |  |  | |  |
| BMI | 1 | Underweight (<18.5) | | 187 | BMI (WHD010, WHD020) | Body Mass Index (BMI) | |  |
|  | 2 | Normoweight (18.5 : 24.99) | | 4990 |  |  | |  |
|  | 3 | Overweight (25 : 29.99) | | 7304 |  |  | |  |
|  | 4 | Obesity (30 : -) | | 8446 |  |  | |  |
| Alcohol | 1 | Never drink | | 10405 | ALQ | Alcohol drinking status | |  |
|  | 2 | Former drinker | | 3477 |  |  | |  |
|  | 3 | Current drinker | | 5677 |  |  | |  |
| Smoking | 1 | Never smoke | | 14532 | SMQ20 | Ever smoke 100 cigarettes? | |  |
|  | 2 | Former smoker | | 585 | SMQ40 | Do you now smoke? | |  |
|  | 3 | Current smoker | | 7133 |  |  | |  |
| CV Risks | 0 | No risks | | 18090 | MCQ160C | Ever told you had coronary heart disease | |  |
|  | 1 | One risk | | 2355 | MCQ160D | Ever told you had angina/angina pectoris | |  |
|  | 2 | Two risks | | 974 | MCQ160E | Ever told you had heart attack | |  |
|  | 3 | Three risks | | 480 | MCQ160F | Ever told you had a stroke | |  |
|  | 4 | Four or more risks | | 249 | DIQ010 | Doctor told you have diabetes | |  |
|  |  |  | |  | LBXTC | Total Cholesterol (mg/dL) | |  |
|  |  |  | |  | HYPE | Derived measure | |  |
| SES |  | Mean=2.59 | | 19899 | INDFMPIR | Poverty income ratio | |  |
| Exercise | 0 | A Lot | | 2006 | PAQ650 | Vigorous recreational activities | |  |
|  | 1 | Moderate | | 7064 | PAQ665 | Moderate recreational activities | |  |
|  | 2 | Little or No | | 12973 |  |  | |  |
| Depression | 0 | Very high | | 901 | DPQ010 to DPQ100 | | |  |
|  | 1 |  | 1160 | |  |  | |  |
|  | 2 |  | 3073 | |  |  | |  |
|  | 3 | No depression | 16107 | |  |  | |  |

Note: BMI: Body Mass Index; SES: Socioeconomic Status; CV: cardiovascular.
